# Purification and characterization of the enzymes involved in nicotinamide adenine dinucleotide degradation by *Penicillium brevicompactum* NRC 829

**DOI:** 10.1007/s13205-015-0349-7

**Published:** 2016-01-21

**Authors:** Thanaa Hamed Ali, Dina Helmy El-Ghonemy

**Affiliations:** Department of Microbial Chemistry, National Research Centre, 33 EL Bohouth St., Dokki, 12622 Egypt

**Keywords:** NAD degradation, *Penicillium brevicompactum* NRC 829, Alkaline phosphatases, Deaminase, Glycohydrolase

## Abstract

The present study was conducted to investigate a new pathway for the degradation of nicotinamide adenine dinucleotide (NAD) by *Penicillium brevicompactum* NRC 829 extracts. Enzymes involved in the hydrolysis of NAD, i.e. alkaline phosphatase, aminohydrolase and glycohydrolase were determined. Alkaline phosphatase was found to catalyse the sequential hydrolysis of two phosphate moieties of NAD molecule to nicotinamide riboside plus adenosine. Adenosine was then deaminated by aminohydrolase to inosine and ammonia. While glycohydrolase catalyzed the hydrolysis of the nicotinamide-ribosidic bond of NAD+ to produce nicotinamide and ADP-ribose in equimolar amounts, enzyme purification through a 3-step purification procedure revealed the existence of two peaks of alkaline phosphatases, and one peak contained deaminase and glycohydrolase activities. NAD deaminase was purified to homogeneity as estimated by sodium dodecyl sulphate-polyacrylamide gel electrophoresis with an apparent molecular mass of 91 kDa. Characterization and determination of some of NAD aminohydrolase kinetic properties were conducted due to its biological role in the regulation of cellular NAD level. The results also revealed that NAD did not exert its feedback control on nicotinamide amidase produced by *P. brevicompactum*.

## Introduction

Increasing evidences have indicated that NAD and NADH play critical roles not only in energy metabolism, but also in several cellular functions including calcium homeostasis, gene expression, ageing, immunological mechanisms and cell death (Leonarda 2008). Nicotinamide adenine dinucleotide (NAD) and its reduced form NADH are essential cofactors for many redox biocatalysts. Because these cofactors are consumed in stoichiometric amounts, whole-cell biocatalysts have been routinely employed to reduce the costs. To further improve the efficacy of redox biocatalysts, it is essential to maintain the stability of nicotinamide cofactors, for which it is necessary to block degradation pathways for NAD (H). While the biosynthesis of NAD (H) has been well studied, it is less understood how NAD (H) are degraded (Wang et al. 2014).

Among studies on NAD degradation by non-pathogenic microorganisms, filamentous fungi are found to be particularly interesting because of their easy cultivation, high production of extracellular enzymes with large industrial potential. Besides, fungal enzymes are more resistant to harsh climatic conditions than other sources. These enzymes are applied in the industrialization of chemical and biomedical products (Guimarães et al. 2006). As newly discovered eukaryotic NAD precursors, NR and NaR have the potential to be important mammalian nutritional supplements and/or drugs (Ma et al. 2007; Bogan and Brenner 2008). Alkaline phosphatases involved in NAD degradation which cleave NAD at the phosphate linkage to produce ADP and nicotinamide riboside have been characterized earlier in our laboratory from *A. niger* (Elzainy and Ali 2000), *A. terreus* (Elzainy and Ali 2003, 2005). Subsequently, alkaline phosphatase splits ADP to AMP plus free inorganic phosphate. AMP was further cleaved by the same enzyme to produce adenosine plus Pi. Non-specific degradation of NAD to nicotinamide riboside was detected in *Escherichia coli* (Wang et al. 2014). The applications of alkaline phosphatase isolated from different sources are reviewed with special reference to the macromolecular structure and action mechanism of the enzyme in the reactions of phosphomonoester hydrolysis. The practicality of alkaline phosphatase as a helpful tool in conducting enzyme-linked immunoassays is demonstrated (Zueva et al. 1993).

N-glycosidic linkage of the released nicotinamide riboside was hydrolytically cleaved by glycohydrolase from the same organism producing nicotinamide (Nm) and free ribose. A glycohydrolase is highly specific for NMN. NAD, NADP, nicotinic acid-adenine dinucleotide, nicotinamide riboside catalyses (Imai 1979). On the other hand, studies on NAD+ biosynthesis originate from the involvement of NAD+ in skin cancer prevention mechanisms that include enhancing DNA repair and preventing photo-immune suppression. In addition, NAD+ serves as a cofactor for energy generation used in cornified epidermal barrier formation and release of agents with protective effects on skin (Leonarda 2008).

Adenosine aminohydrolase (ADA) participates in the purine metabolism, whereas it degrades either adenosine or 2′-deoxyadenosine producing inosine or 2′-deoxyinosine, respectively (Kocic et al. 1991). The physiological function of ADA is critical in controlling the effects of these metabolites on immunological, neurological and vascular systems. ADA is also involved in the development of *T. lymphocytes* band as it is evident from the fact that ADA deficient animals suffer from B and T lymphopaenia (Ray and Sharma 2002; Ashok et al. 2008). Serum ADA levels increase in pancreatic disorders, especially in pancreatic cancer; therefore it may be used as a serum marker for the diagnosis of pancreatic cancer (Bi et al. 2007). The evidence of high ADA activity during rapid and stimulated growth of normal tissues is of importance in making a fully functional purine salvage pathway possible (Seiler 2004). Adenosine deaminase of *Aspergillus oryzae* (Ali et al. 2012, 2014) has been characterized in our laboratory. Both the adenosine and by-products of the NAD-consuming enzymes such as NMN and Nm can be recycled back to NAD (Sorcil et al. 2010; Gazzaniga et al. 2009). Therefore, the present research focused on studying in detail the degradation of NAD+ by *Penicillium brevicompactum* NRC 829 extracts. Besides, NAD aminohydrolase was purified to homogeneity and some of kinetic properties were characterized.

## Materials and methods

### Chemicals

Adenosine, adenine, inosine, cytosine, guanine, cytidine, adenosine 5′-monophosphate (AMP), guanosine 5′-monophosphate (GMP), NAD and cytidine 5′-monophosphate (CMP) were purchased from Sigma Chemical Company. Nicotinamide and nicotinic acid were purchased from Merck. DEAE-Sephadex A-25 and Sephadex G-100 were from Pharmacia Fine Chemical. All other reagents were prepared in Microbial Chemistry Department, National Research Centre.

### Microorganism

*Penicillium brevicompactum* NRC 829 was obtained from culture collection of Microbial Chemistry Department, National Research Centre, Dokki, Egypt.

### Medium

The selected fungal strain was grown and maintained on slants of solid modified Czapek Dox’s medium containing g l^−1^ distilled water: glucose, 30; NaNO_3_, 2.0; KH_2_PO_4_, 1.0; MgSO_4_·7H_2_O, 0.5; KCl, 0.5 and agar, 20.

#### Preparation of *Penicillium brevicompactum* extracts

The 4-day-old mats, grown on liquid modified potato dextrose medium, containing per litre 300 g of potato and 20 g dextrose at 28 °C, were harvested by filtration, washed thoroughly with distilled water and blotted to dry with absorbent paper. The mats were then ground with cold washed sand in a chilled mortar and extracted with cold distilled water. The slurry obtained was centrifuged at 1, 5229×*g* for 10 min and the supernatant was used as the crude enzyme preparation.

### Enzyme assay

Alkaline phosphatase was determined according to the method described by Heninone and Lahti (1981), summarized as follows: the stock solutions consist of 10 mM (NH_4_)_6_Mo_7_O_24_.4H_2_O, 1 M citric acid and 5 N H_2_SO_4_, all in distilled water and stable at least for several weeks at 25 °C. Acetone–acid–molybdate (AAM) solution was prepared daily by mixing 1 vol of ammonium molybdate solution with 1 vol of 5 N H_2_SO_4_ and 2 vol of acetone. Inorganic phosphate determination: into a test tube containing 0.5 ml of sample, 4 ml of AAM solution was added. The contents were mixed carefully with a vortex mixer and 0.4 ml of 1 M citric acid was pipetted into each tube. After mixing, the yellow colour observed was measured at 390–420 nm. A sample with no added Pi was used as a blank. Adenosine deaminase activity was determined as ammonia according to the method described by Vogel (1961). Briefly, 0.5 ml of purified enzyme solution was mixed with 0.5 ml of Tris–acetate buffer (0.2 M, pH 5.0) containing 5 µM NAD. The mixture was incubated at 40 °C for 30 min and the reaction was terminated by the addition of 0.5 ml of Nessler’s reagent. The yellow colour formed was determined spectrophotometrically by measuring its absorbance at 450 nm. Nicotinamide riboside glycohydrolase was determined by the method described by Shin et al. (1999). Unit of enzyme activity was defined as the amount of enzyme required to produce 1 µM of product, i.e. NH_3_ or Pi or ribose, per min under the standard assay conditions.

### Protein determination

Protein concentration was determined according to Lowry et al. (1951), using bovine serum albumin (BSA) as a standard. The protein content of the purified enzyme fractions was determined by the UV absorbance according to the method of Schleif and Wensink (1981).

### Thin layer chromatography (TLC) analysis

Enzymatic reaction products were assayed by TLC Silica gel 60254 (Aluminium sheet) (20 × 20 cm) according to a procedure presented by Kemmer et al. (2001) and Lee et al. (2000). Reactions were done at 40 °C for 2 h in 80 µl of Tris–HCl (pH 7.5) that contained 5 µM NAD and 0.4 mg ml^−1^ enzyme of *P. brevicompactum*. Reactions were terminated by the addition of 5 µl of 2 M HCl and centrifuged at 12,000×*g* for 10 min. Samples were spotted onto TLC sheet, developed in a, *n*-butanol:acetone:acetic acid (glacial):ammonia (5 %):water (45:15:10:10:20) mixture (Smith and Seakins 1976) and visualized under UV at 254 nm.

### Separation and partial purification of NAD degrading enzymes

#### Acetone fractionation

Cold acetone (−20 °C) was added to the crude extract at various concentrations of 0–30, 30–60, 60–90 %, respectively. The suspension formed was centrifuged at 10,000 rpm for 15 min at 4 °C. The precipitate formed was collected and dissolved in a minimal volume of Tris–acetate buffer, pH 7.0 (0.02 M).

#### Dialysis

The sample obtained after acetone fractionation was dialysed for 3 h against cold distilled water at 7 °C using a dialysis bag (Size 3, 20/32–15.9 mm, Medicell International Ltd).

#### DEAE-Sephadex A-25 chromatography

The dialysed partially purified enzymes were loaded onto a DEAE-Sephadex A-25 column (1.0 × 45 cm) that was pre-equilibrated with the same buffer (0.1 M). Elution was carried at room temperature by batch-wise additions of 50 ml portions of increasing molarities (0.0–0.5 M) of sodium chloride solutions in 0.1 M Tris–acetate buffer pH 7, at flow rate 20 ml h^−1^. Fractions of 5.0 ml were collected and analysed for protein and the enzyme activities were determined as described previously. Fractions with high enzyme activities were pooled together, dialysed against the same buffer and finally concentrated by lyophilization (−50 °C) for further analysis.

#### Sephadex G-100 gel filtration

The lyophilized concentrated solution was further loaded onto Sephadex G-100 column (46 × 2.0 cm), which was equilibrated in 0.1 M Tris–acetate buffer pH 7.0. The enzyme was eluted from the column using the same buffer at a flow rate of 30 ml h^−1^, at room temperature (25 °C). The fractions were analysed for protein, Pi, NH_3_ and ribose determination.

Specific activity was expressed as µmol NH_3_ or Pi or ribose liberated from substrate (NAD) per mg protein per min. Appropriate control reaction mixtures, i.e. the enzyme source or the substrate was used as blanks throughout the work. All the experiments cited in this work were conducted in triplicate and the mean values were reported and all the results recorded were reproducible.

### Molecular mass determination by SDS-PAGE

SDS-gel electrophoresis technique was used to detect the purity of enzyme and to determine molecular mass of the purified enzyme from *P. brevicompactum* NRC 829 according to the method described by Laemmli (1970), by using the following proteins which were used as molecular weight standards (Fermentas) (spectra TM multicolor broad range protein ladder): 250, 150, 100, 70, 50, 40, 30, 20 and 10 kDa.

### Extent of NAD degradation by *P. brevicompactum* extracts

The experiment aimed to determine the extent of NAD degradation when *P. brevicompactum* extracts were incubated with NAD as a substrate, at optimum pH and temperature. The release Pi, NH_3_ and ribose were colourimetrically analysed at different time intervals over a period of 3 h.

### Determination of optimal pH, temperature and stability

The optimal pH of the purified enzymes was determined by performing the enzyme assays in the appropriate buffers: KCl–HCl (pH 1.0–2.0); Tris–acetate (pH 3–6.0); Tris–HCl (pH 6.0–9.0) and carbonate–bicarbonate (pH 9.0–10.0). Temperatures of the enzyme were determined by performing the enzyme assays within the temperature range of 20–80 °C. The thermal stability of the purified enzymes was examined by measuring the residual activity after incubating the enzyme at each desired temperature for 30 min.

### Influence of different compounds on the purified enzyme activity

The purified enzymes were pre-incubated with different compounds (10 mM) at 40 °C for 30 min in Tris–acetate buffer (50 mM, pH 6.0). The metal ions and some modulators were Fe^2+^, Fe^3+^, CO^2+^, Ca^2+^, Mg^2+^, Zn^2+^ and Mn^2+^. The enzyme activities was determined as described above using NAD as substrate. The enzyme activity without any agent was taken as 100 %.

### Substrate specificity

Substrate specificity was investigated by replacing NAD in the assay mixture with an equal concentration of the representative phosphorylated, aminated riboside compounds.

#### Kinetics of *P. brevicompactum* NAD aminohydrolase

Kinetic parameters of *P. brevicompactum* aminohydrolase against NAD were determined in triplicates at 40^◦^C for 30 min. Various concentrations of NAD+ (1.5–20 μM) in 50 mM Tris-acetate buffer (pH 6.0) were used in the reaction mixture. Released ammonia was assayed discontinuously as previously described.

## Results and discussion

### Extent of NAD degradation

**Fig. 1:**
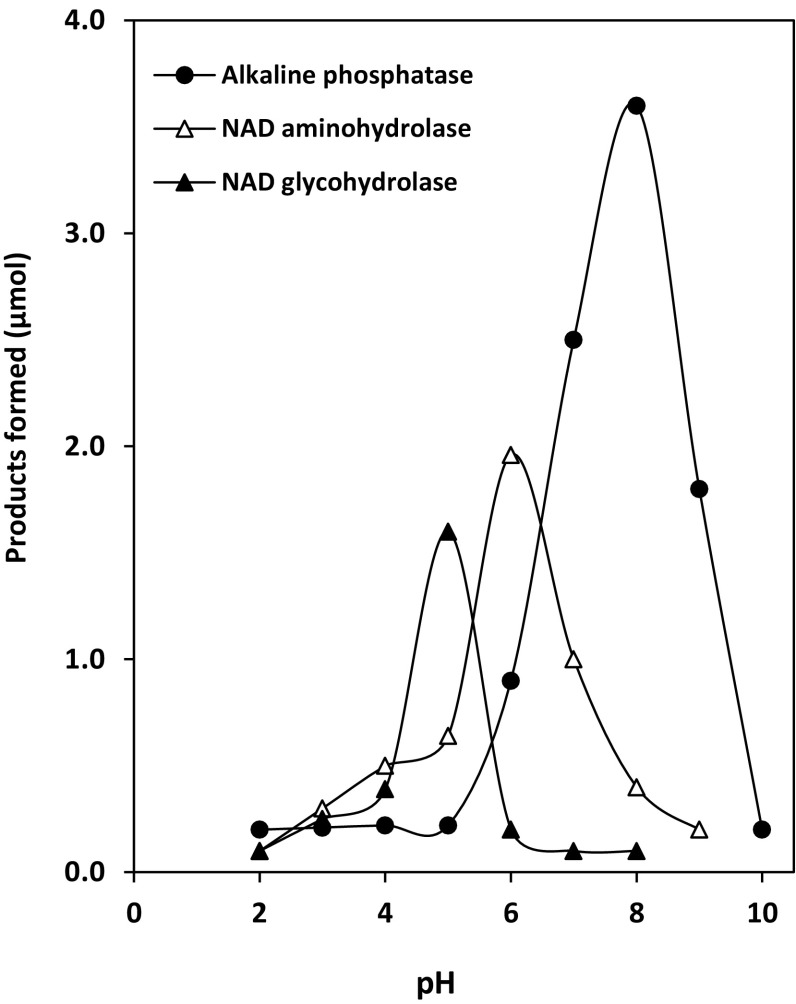
Optimal pH of NAD degrading enzymes. The reaction mixture contained the following: substrate, NAD, 5 µmol; buffers of various pHs: 80 µmol (pH 3–6) citrate-buffer, (pH 6–9) Tris–acetate, (pH 9–10) carbonate–bicarbonate buffer; temp., 50 °C; protein extracts, 2.5 mg

*Penicillium brevicompactum* NRC 829 extracts were incubated with NAD at different pH values. The results observed indicated that Pi and NH_3_ were optimally liberated from NAD at pH 8 and pH 6.0, respectively, while reducing sugar was optimally detected at pH 5.0. In addition, it was also found that the release of phosphate in the reaction adjusted at pH 8 was faster than the reactions at pH 6 or pH 5 (Fig. 1). Complete phosphate hydrolysis could be detected in the reaction mixtures after 3 h of incubation. The observed properties of phosphatases isolated from *P. brevicompactum* were similar to fungal phosphatases produced by *A. niger* (Elzainy and Ali 2000), *A. terreus* (Elzainy and Ali 2003) and *A. oryzae* (Ali et al. 2012). These enzymes are similar in being orthophosphate- non repressible enzymes and being not true phosphomonoesterases. This last property was based on their abilities to catalyse the hydrolysis of pyrophosphate linkage of ADP and the internal ester linkage between NR and ADP, in addition to the true phosphate ester linkages of NAD, ADP and AMP. At the end of the incubation period, almost all the phosphate links of the dinucleotide molecule were cleaved, while about 40 % either of the amido- or the amino-linkages were hydrolysed. However, this percentage may represent the sum of percentages of hydrolysed amido- and amino-bonds. Also the reducing power of 32 % may be formed as a result of the cleavage of N-glycosidic bond in adenosine or nicotinamide riboside.

NAD degradation pathway by the extracts of *P. brevicompactum* was summarized as shown in the following diagram.
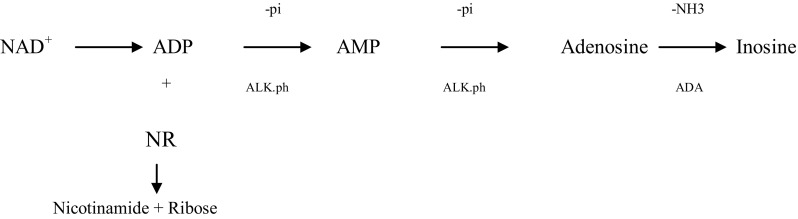


### Identification of products and intermediates of NAD degradation

**Fig. 2:**
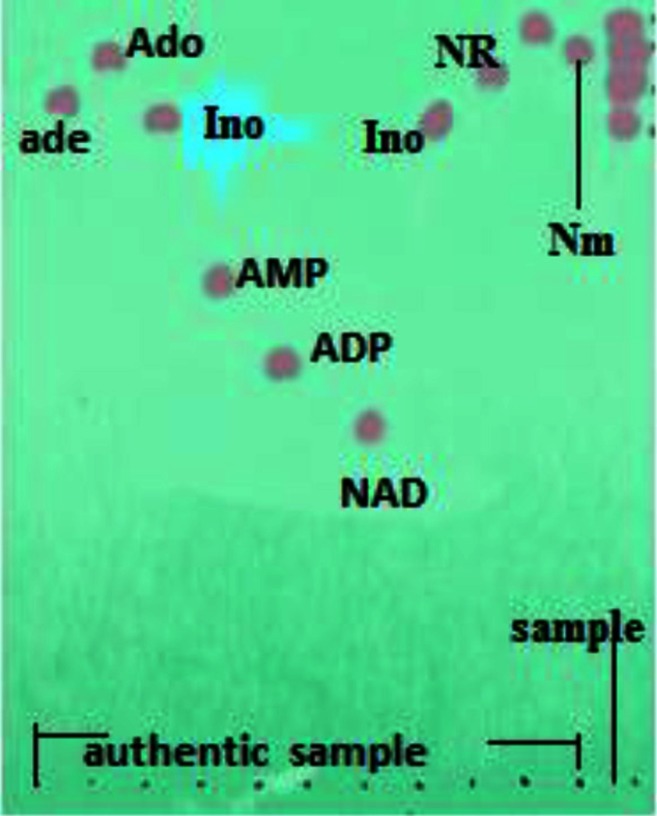
TLC analysis of products from NAD degradation activity of *P. brevicompactum* aminohydrolase. *Right lane* was the sample of reaction mixture containing 5 µM NAD being treated at 50 °C for 2 h by aminohydrolase at an initial concentration of 20 µg. Concentrations of chemical standards were 2 mM. Abbreviations of authentic: NAD, nicotinamide adenine dinucleotide; AMP, adenosine 5′-monophosphate; ADP, adenosine 5′-diphosphate; Ado, adenosine; Ade, adenine; Ino, inosine; Nm, nicotinamide; NR, nicotinamide riboside

Analysis of the reaction mixture, in which inorganic phosphate, ribose and ammonia were formed, showed the extent of NAD degradation by *P. brevicompactum* enzymes. TLC clearly indicated that NAD was consumed, but there was apparently no accumulation of either ADP or AMP. Instead, the presence of NR, Nm, adenosine and inosine was evident for those lanes. These data showed that the end product NAD dephosphorylation was nicotinamide riboside (NR) and adenosine. Subsequently, adenosine was partially deaminated to inosine, while nicotinamide riboside was cleaved to nicotinamide and ribose. Adenosine, inosine, nicotinamide riboside and nicotinamide were located on TLC sheet as the final products (Fig. 2). These results are in agreement with that reported for NAD degradation in the extracts of *A. niger* (Elzainy and Ali 2000), *A. terreus* (Elzainy and Ali 2003) and *A.* *oryzae* (Ali et al. 2012); however, the present research differs regarding the formation of nicotinamide which formed due to the existence of NAD glycohydrolase, while, in case of other fungal strains, nicotinamide riboside remained without further degradation. The detection of inosine and adenosine in the chromatographic analysis demonstrated the partial deamination of adenosine. This result is in congruent with the result previously investigated in *A.* *oryzae* extracts (Ali et al. 2012).

### Separation of NAD deaminating and NAD glycohydrolase activities from NAD dephosphorylating activity

**Fig. 3:**
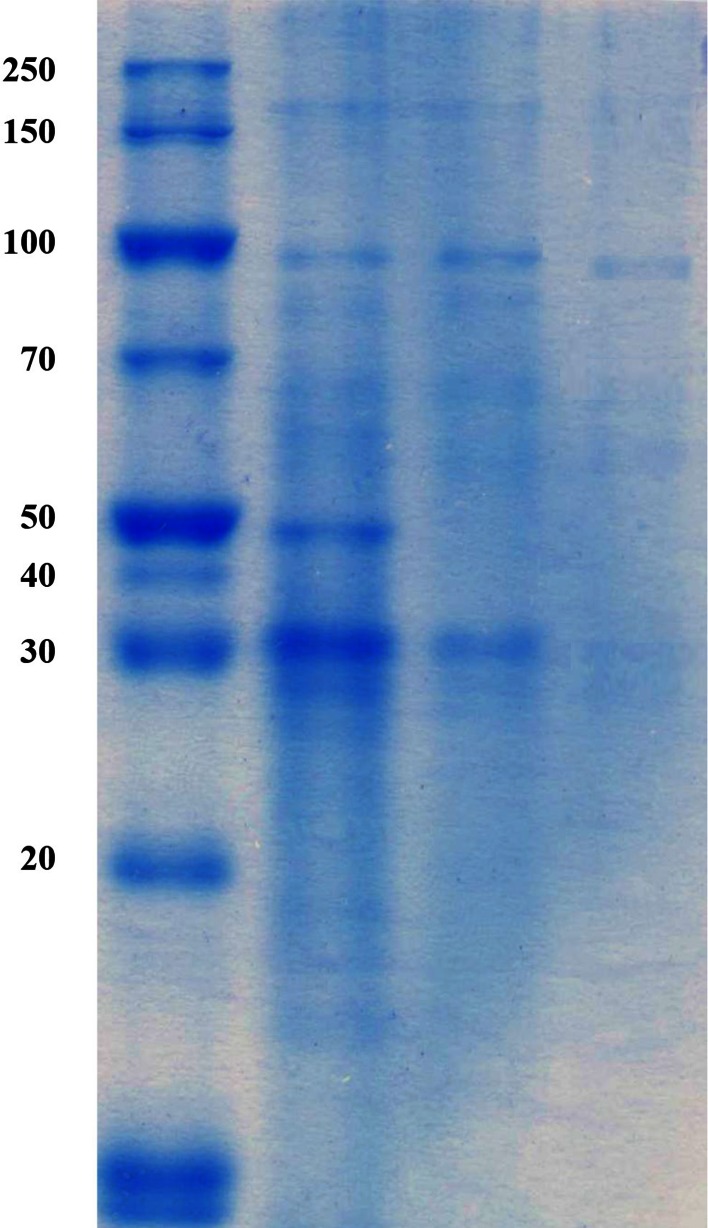
Electrophoretic analysis of *Penicillium brevicompactum* NRC 829 NAD aminohydrolase. From *left* to *right*: *lane 1* molecular mass markers, *lane 2* fractional precipitation by chilled acetone, *lane 3* partial purified NAD aminohydrolase on DEAE-Sephadex A-25, *lane 4* purified NAD aminohydrolase on Sephadex G-100

The data presented in Table 1 showed the purification summary of the three enzymes responsible for NAD degradation. During all purification steps, the activity of alkaline phosphatase was detected in protein fractions with two peaks; this result indicates the existence of two molecular forms of alkaline isozymes. In addition, the activities of NAD glycohydrolase and NAD deaminating were recorded in one peak. The separation of NAD glycohydrolase and NAD deaminase was illustrated by Sephadex G-100 column chromatography described under ‘‘Materials and Methods’’ section. The enzyme was purified to homogeneity. SDS-PAGE showed that it had a molecular mass of 91 kDa (Fig. 3). In this respect, the molecular masses of 94 and 85 kDa were reported for the enzymes produced by *A.* *oryzae* and *A. fumigatus* 4 as investigated by Ali et al. (2014) and Yoshimune et al. (2005), respectively. On the other hand, the purified enzyme from *A. oryzae* was found to exhibit a smaller molecular mass of 14.5 kDa as reported by Rosinova et al. (1978).

**Table 1:** Purification of the NAD degrading enzymes from *P. brevicompactum* NRC 829

Purification steps	Total activity (units)	Protein (mg)	Sp. activity	Recovery (%)	Purification fold
Alkaline phosphatase					
Crude extracts	600	390	1.5	100	1
Acetone fraction	562	150	3.7	93	2.5
DEAE-Sephadex A25					
ALK1	230	3.0	60.6	38	51.1
ALK2	200	2.8	71.4	33	47.6
NAD aminohydrolase					
Crude extracts	450	390	1.1	100	1
Acetone fraction	400	150	2.6	88	2.2
DEAE-Sephadex A25	150	5	30	30	27.3
Sephadex G-100	100	2	50	22	41
NAD glycohydrolase					
Crude extracts	290	390	0.75	100	1
Acetone-fraction	245	150	1.6	84	2.1
DEAE-Sephadex A 25	130	5	27	44	36
Sephadex G-100	76	1.9	40	26	50

### Characterization of purified NAD deaminase

#### Optimal pH and temperature

**Fig. 4:**
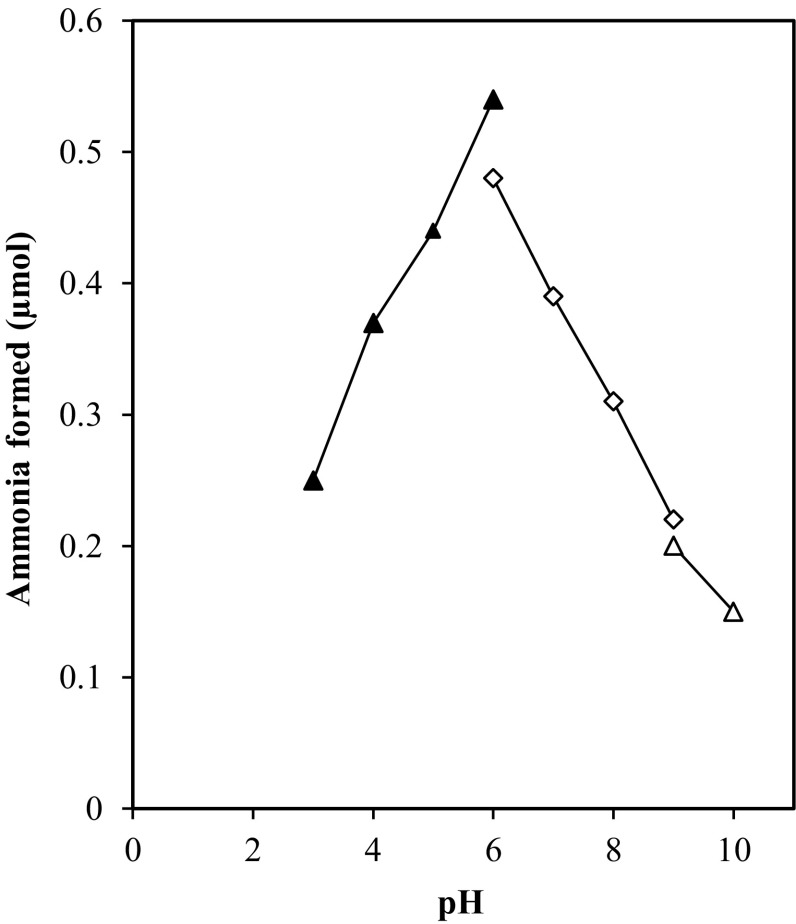
pH dependence of the purified deaminase activity

**Fig. 5:**
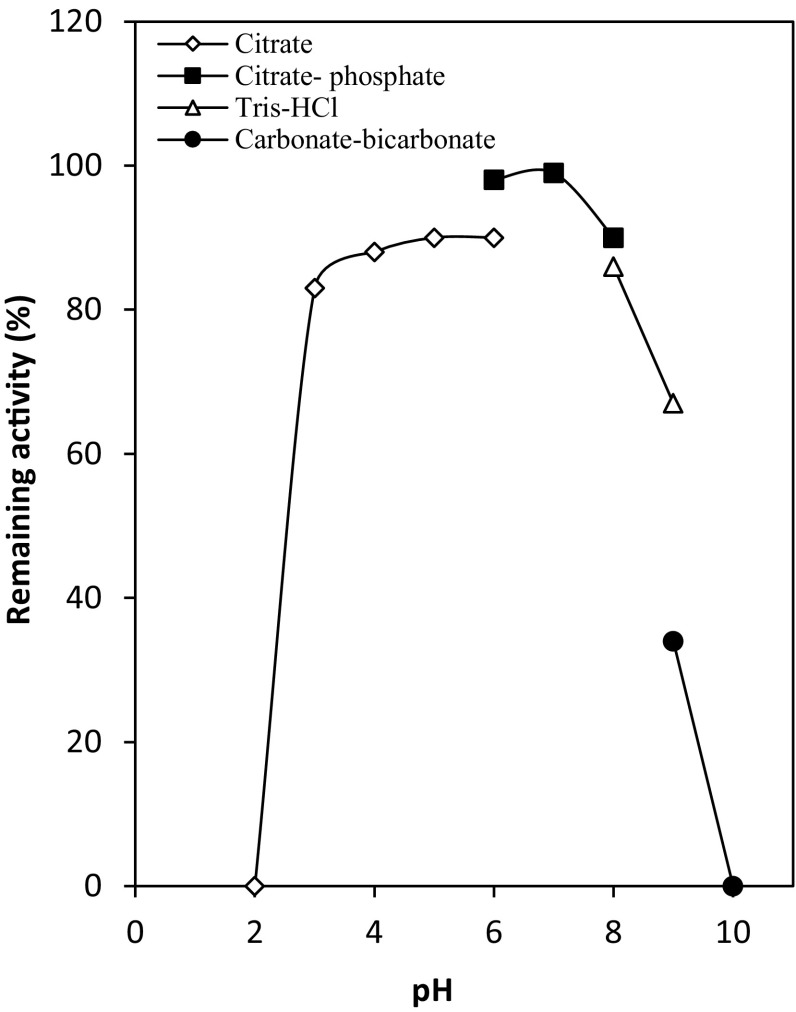
pH stability of the NAD aminohydrolase. The enzyme was stored in buffers of various pHs (100 mM) at 50 °C for 30 min, and the residual activities were measured

**Fig. 6:**
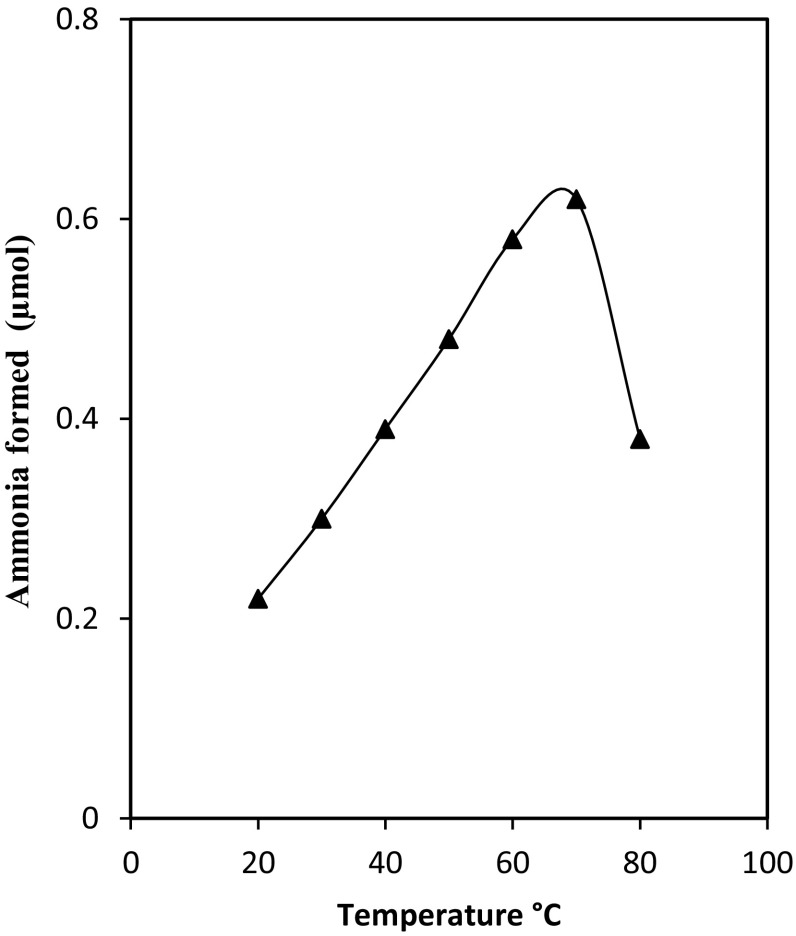
Effect of temperature on purified deaminase

The effect of pH on deaminase activity was examined over a wide pH range of 3.0–10.0. The results illustrated in Fig. 4 demonstrated that the deaminase displayed optimal activity at pH 6.0. The enzyme was stable when pre incubated in the pH range of 6.0 to 8.0, while 50 and 55 % of its original activity were observed at pH_s_ 4.0 and 9.0, respectively (Fig. 5). This is similar to the optimal pH (5–7) of adenosine-phosphate deaminase of *A. fumigatus* (Yoshimune et al. 2005), NAD deaminase produced by *A.* *oryzae* (Ali et al. 2014). At pH 8.0, the deaminase loses 40 % of its activity as *A.* *oryzae* NAD deaminase, which had its optimum activity at pH 5 and abroad pH stability. The optimal temperature for the deaminase activity was in the range of 50–70 °C (Fig. 6). This is about 10–20 °C higher than optimum temperature of the adenosine-phosphate deaminase of *A. fumigatus* (Yoshimune et al. 2005) and NAD deaminase *A.* *oryzae* (Ali et al. 2014), respectively. The enzyme was found to thermostable up to 70 °C; the results also showed that 80 and 88 % of its original activity still remained at 50 and 60 °C, respectively, after 30 min of incubation (Data not shown). The enzyme purified from *P. brevicompactum* was considerably more thermostable with higher specific activity than deaminase of *A. fumigatus* and *A.* *oryzae*.

#### Effect of various agents on the purified enzyme activity

In the present study, results in Table 2 clearly showed that the enzyme was enhanced about 30 % by Zn^2+^ (1 mM), while no significant enhancement of the enzyme activity was reported by Ca^2+^, Co^2+^, Fe^2+^, Mg^2+^, Mn^2+^ and Fe^3+^. In addition, the present data revealed that the enzyme activity was not inhibited by iodoacetate, used at 10 mM final concentration, which clearly indicates the absence of evidence for the involvement of an SH group(s) in the catalytic action of enzyme. The effect of EDTA, known as a metal chelating agent, on enzyme activity was tested to find out whether this enzyme is a metalloenzyme or not. It was found that the addition of EDTA (10 mM) to the reaction mixture did not inhibit enzyme activity indicating that NAD deaminase is not a metalloenzyme. This property closely resembles that of a nonspecific NAD aminohydrolase produced from *A. oryzae* (Ali et al. 2014); whereas the enzyme was slightly activated by addition of Na^+^ and K^+^, while inhibited by addition of Mn^2+^, Ag^+^, Hg^2+^, and Cu^2+^. In this concern, Yoshimune et al. (2005) reported that the adenosine-phosphate deaminase produced by *A. fumigatus* was inhibited by 38 % in the presence of Cu^2+^ (1 mM), while no significant enhancement of the enzyme activity was reported by Ba^2+^, Ca^2+^, Co^2+^, Fe^2+^, Mg^2+^, Mn^2+^, Ni^2+^, Sn^2+^, Zn^2+^ and Fe^3+^. Yoshino and Murakami (1980) reported that the baker’s yeast AMP deaminase was activated in the presence of various divalent cations.

**Table 2:** Effect of metal ions and various chemical reagents on the enzyme activity

Reagents (1 mM)	Relative activity (%)
None	100
Zn^2+^	132
Ca^2+^	92
Co^2+^	94
Fe^2+^	95
Mg^2+^	90
Mn^2^	91
Fe^3+^	89
EDTA	92
Iodoacetate	93
Mercaptoethanol	96

#### Substrate specificity

The substrate specificity of purified NAD deaminase was examined and the results were tabulated in Table 3, from which it was found that the enzyme was active toward adenine and various adenine derivatives (adenosine, AMP, ADP, NAD, ATP and NAD) while no activity was detected towards nicotinamide or nicotinamide riboside as a nonspecific NAD aminohydrolase from *A. oryzae* (Ali et al. 2014). In contrast, *A.* *terreus* NAD deamidase catalysed amide group of the intact NAD molecule and could also cleave the amide group from NMN, NR, Nm, glutamine and asparagine (Elzainy and Ali 2005). The microbial AMP-deaminating activity was catalysed by adenosine-phosphate deaminase (EC 3.5.4.17) and adenylic acid deaminase (EC 3.5.4.6) (Yoshimune et al. 2005). The enzyme from Takadiastase (*A. oryzae*) was identified as a nonspecific adenosine deaminase (EC 3.5.4.4, adenosine aminohydrolase) by Minato et al. (1965); this enzyme deaminated various adenosine derivatives (3′-AMP, 5′-AMP, and NAD), but not adenine or NADP. The enzymes from *A. oryzae* and *A. fumigatus* 4 were different in substrate specificity, as described above. The enzyme from *S. aureofaciens* catalyses the deamination of adenosine, 5′-AMP, ADP and ATP, but not that of adenine (Rosinova et al. 1978). The enzyme from *Streptomyces* sp. is active towards adenosine, 2′-deoxyadenosine, 3′-AMP, 5′-AMP and cAMP (Jun et al. 1991), but the enzyme activities towards 3′-AMP, 5′-AMP and cAMP were 4– 8 % of those of adenosine, indicating that *Streptomyces* sp. enzyme is classified as adenosine deaminase. As described above, the substrate specificities of microbial adenosine-phosphate deaminases are different depending on the origin of the enzymes.

**Table 3:** Kinetic parameters of *P. brevicompactum* aminohydrolase

Substrate	*K*_m_ (µM)	*K*_cat_ (s^−1^)	*K*_cat_/*K*_m_ (M^−1^ s^−1^)
NAD	5.2	10.08	1.93
AMP	8.33	8.12	0.98
ADP	6.25	0.93	0.207
Adenosine	4.5	11.2	1.79
Nicotinamide riboside	ND	ND	ND

*ND* not detected

#### Kinetic parameters of NAD aminohydrolase

Kinetic parameters of *P. brevicompactum* aminohydrolase catalysis were determined using NAD, ADP, AMP and adenosine, as substrates (Table 3). While *K*_m_ values were close for all of these substrates, *K*_cat_ values for AMP and ADP were orders of magnitude higher than those for NAD and adenosine, indicating that aminohydrolase had substantially higher catalytic efficiency for ammonia hydrolysis. It should be pointed out that the *P. brevicompactum* had the highest NAD and adenosine aminohydrolase activity compared to the enzyme produced by *A. oryzae* (Ali et al. 2014). The *K*_m_ values for NAD (5.2 µM) and adenosine (4.5 µM) of *P. brevicompactum* were substantially lower; indicating aminohydrolase has much stronger binding affinity towards NAD and adenosine compared to other enzymes for NAD degradation.

## Conclusion

The present work demonstrated the occurrence of two alkaline phosphatases, aminohydrolase and glycohydrolase in *P. brevicompactum* NRC 829. These enzymes are involved in NAD degradation. Purification and separation showed high aminohydrolase activity with the catalytic efficiencies for hydrolysis of NAD and adenosine at 1.9 and 1.8 μM^−1^ s^−1^, respectively. These results significantly enriched our understanding on NAD metabolism and should facilitate many applications including designing redox biocatalysts.
